# Assessing the validity of tuberculosis surveillance data in California

**DOI:** 10.1186/1471-2458-6-217

**Published:** 2006-08-25

**Authors:** Joan E Sprinson, Elizabeth S Lawton, Travis C Porco, Jennifer M Flood, Janice L Westenhouse

**Affiliations:** 1 TB Control Branch, Division of Communicable Disease Control, California Department of Health Services, 850 Marina Bay Parkway, Building P, 2nd floor, Richmond, CA 94804–6403, USA; 2 Center for Infectious Disease Preparedness, University of California at Berkeley School of Public Health, 1918 University Avenue, 4^th ^floor, MC 7350, Berkeley, CA 94720–7350, USA; 3 Box 1211 – EXCP, University of California of San Francisco, San Francisco, CA 94143–1211, USA

## Abstract

**Background:**

The Centers for Disease Control and Prevention (CDC) convened a workgroup to revise the tuberculosis (TB) case report in the United States of America (U.S.). The group proposed substantial revisions. Study objectives were to systematically assess the validity and completeness of reported TB case surveillance data in California and to inform TB case report revision process.

**Methods:**

A sample of 594 cases was retrospectively selected from the cohort of all TB cases reported during 6/1/96-5/31/97 to the State TB Registry. Cases, stratified by treatment outcome, were randomly sampled within each outcome category. Data for 53 variables were abstracted from each case's public health medical record and compared to data recorded on the TB case report. Using the medical record as the "gold standard," estimates were developed for 1) concordance, sensitivity, and positive predictive value of reported data for categorical variables; 2) the absolute mean difference between the two information source for date variables; and 3) the completeness of data on the case report and in medical record.

**Results:**

At least 90% of the values for 35 (79.5%) categorical variables submitted on the TB case report form were identical to values in the medical record. Concordance between data on the case report and medical record was lower for the remaining nine (20.5%) categorical variables: status of abnormal chest x-ray (46.8%); directly observed therapy (48.6%); smear result for tissue or body fluid other than sputum (49.2%); type(s) of tissue or body fluid for smears and cultures other than sputum (76.4% and 73.9% respectively); provider type (73.4%); occupation (84.4%); sputum culture conversion (85.4%); and sputum smear result (89.6%). Case report data were more complete than data in the medical record; 2.9% versus 9.8% of data were missing/unknown, respectively.

**Conclusion:**

For most variables examined on the TB case report, data validity was excellent, indicating a robust surveillance system. However, lower data quality was noted for a small number of variables primarily impacting treatment adherence, including assessment and planning; advocacy; allocation and garnering of resources; and research. The study provides compelling evidence supporting the CDC workgroup's proposed revisions to the TB case report.

## Background

Tuberculosis (TB) surveillance data are essential to evaluate the effectiveness of TB control programs, identify deficiencies, and design and assess interventions. Surveillance data are also critical in advocacy efforts and in garnering and allocating resources. For these reasons, the World Health Organization's (WHO) enhanced DOTS initiative [1-3] and the Centers for Disease Control and Prevention (CDC) emphasize the importance of monitoring and evaluating program performance [4,5]. In order to perform these important functions, it is essential that the right variables are collected and reported in a timely manner, and that these data are valid and complete. Inadequate data quality may impair our understanding of the true epidemiology of TB, compromise core program functions, and undermine our ability to meet program objectives and goals. Although the availability of accurate and complete TB surveillance data varies considerably by region and by country, these data provide an essential tool for local, national, and global efforts to control and eliminate TB.

Since 1985, all 50 states within the United States of America (U.S.) have collected and submitted data for each confirmed TB case to the CDC on the Report of Verified Case of Tuberculosis (RVCT). In January 1993, in response to the TB resurgence in the U.S., CDC increased the length of the RVCT from 1 to 4 pages, the number of questions from 23 to 41, and the number of variables from 37 to 114 [6,7]. The RVCT collects the following categories of information on confirmed TB cases: personal identifier, demographic, social/behavioral risk factor, clinical, treatment, outcome, and case management. The CDC convened a workgroup of local and state TB surveillance directors to examine the need for revisions to the RVCT and accompanying instructions. Over a two year period, the workgroup developed a draft that proposed substantial revisions. At this time, RVCT revisions are still in process. As the draft undergoes review by stakeholders, further revisions are anticipated, with implementation of the revised case report form planned for 2008 (V. Robison, Chief of Surveillance Team, Division of Tuberculosis Elimination, CDC, personal communication; 6/8/06).

In California, a state which for many years has reported over 20% of U.S. TB cases, medical providers report suspected and confirmed TB cases to the 61 local health jurisdictions in which the case resides. Local health departments report confirmed cases, either electronically or manually, to the California TB Registry in the state health department. This reporting occurs: 1) at TB case confirmation; 2) when the health department receives initial drug susceptibility test results (for culture positive cases); and 3) when final treatment outcome information is available, typically 6–18 months after treatment is initiated. Local health departments may also submit updated information to the State TB Registry subsequent to the initial case report submission. After the submission of RVCTs, State Registry staff perform systematic quality control (QC) checks of data and collaborate with local TB registry staff to ensure valid and complete data.

To our knowledge, there are no published studies that have comprehensively assessed the validity and completeness of TB surveillance data in the U.S. or within California. Two U.S. researchers who examined the quality of TB surveillance data for a small number of variables found inaccuracies when comparing information obtained from public health medical records and electronic case reports. In one report, a state health department found substantial differences in the values of ten variables at six local health districts [8]. In another study of causes for delayed treatment completion in three state cohorts, incorrect reporting in one state resulted in misclassification of therapy completion status for 36% of its cases [9].

The primary objectives of this study were to systematically assess the validity and completeness of RVCT data in California and to inform the RVCT revision process.

## Methods

This evaluation of the validity and completeness of TB surveillance data used data from two sources: 1) a case study conducted by the California Department of Health Services in 1999 on adverse outcomes during treatment of California TB patients; and 2) RVCTs submitted to the California TB Registry for cases in this study population. There were 594 cases in the study population, sampled from a cohort of 2,627 eligible cases reported during 6/1/96-5/31/97 to the California TB Registry. Cases in the study population met the following criteria: alive at diagnosis; started TB therapy for culture positive TB disease; resided in one of 18 California counties reporting ≥ 50 cases during the study period; did not move outside California during TB treatment; had drug susceptibility testing results available. A stratified random sample design was used to select cases by reported treatment outcome. The sample consisted of 17.7% of patients who completed treatment, 93.2% of patients defaulting from treatment (defined as patient lost, uncooperative, or refused treatment), and 46.7% of patients who died during treatment reported in the study period. RVCTs from all 18 participating local health jurisdictions were submitted electronically to the California TB Registry.

Study research staff performed an in-depth review of the public health medical record at the local health department for each case in the sample. Because the reviewed records consisted of public health medical records only and were used for public health research, institutional review and patient consent were not required. Information collected included baseline socio-demographic, clinical, and provider characteristics; treatment information, including initial regimens, the number of doses of anti-TB medications, documentation of bacterial conversion of sputum cultures from positive to negative, and case management characteristics such as therapy administration and assessment of risk factors for treatment non-adherence. For patients whose care was managed or co-managed by the health department (approximately two-thirds of the sample), the public health medical record was essentially the same as the patient record. For patients whose TB treatment was managed by private practitioners, the public health medical record (hereinafter referred to as the medical record) was frequently less complete, but typically contained information sufficient for health department oversight and reporting, including socio-demographic, clinical, and treatment information, and documentation of medical oversight and case management activities provided by the health department. Since local health departments use the medical record as the primary information source to complete the RVCT, and all reported information should be contained in this record, we considered it the "gold standard." Study staff did not abstract information from the RVCT if a paper RVCT form was included in the medical record, but all other data pertaining to RVCT variables of interest were abstracted from the medical record.

We used the California-specific RVCT reporting instructions in effect during the study period to determine how information contained in the medical record should have been reported [10]. These instructions, based largely on CDC reporting instructions, contain additional detail for a small number of variables. With minor exceptions, these reporting instructions have remained in effect from the study period until the present.

We assessed the validity of reported data for 53 RVCT variables, consisting of 44 categorical variables and nine dates. Table 1 presents a breakdown of the types of variables, values, and validation measures estimated. We followed the CDC's recommendations for assessing data quality in public health surveillance systems [11]. Specifically, we estimated the sensitivity and the predictive value of the positive (PVP) reported value for each categorical variable. We defined sensitivity as the probability of a positive value on the RVCT given documentation of a positive value in the medical record. We defined the PVP as the probability of a documented positive value in the medical record given a positive value on the RVCT. We also estimated the concordance, as a general measurement of agreement, between data in the medical record and the RVCT. We excluded from the validation analyses all null values and values of 'unknown' and 'not done' on the RVCT and 'not charted' in the medical record.

In order to maximize the comparability of the RVCT and medical record for sputum smear and sputum culture results, we restricted the validation of these variables to results from patients diagnosed with pulmonary TB disease only who did not have positive results associated with smear and/or culture non-sputum specimens. This restriction was necessary because the field study did not distinguish between results from sputum specimens, other types of pulmonary specimens such as bronchial wash or tracheal aspirate, and specimens from non-pulmonary sites, whereas the RVCT distinguishes between sputum and non-sputum specimen results. We also created a variable for site of TB disease based on the sites of smear and culture specimens documented in the medical record and compared this to a variable calculated from reported information regarding TB disease site.

We calculated two validation measures for dates: the absolute mean difference in days (seven dates) or months (two dates) between the RVCT and the medical record.

To assess the completeness of data from the case report and the medical record, we estimated the proportion of missing/unknown data in both data sources for all variables. For nine RVCT variables, values are expected depending on the value of another variable (e.g., a value for type of chest x-ray abnormality or status of chest x-ray is expected only if chest x-ray results are abnormal). For these nine variables, we restricted our estimate of missing/unknown values to instances in which a subsequent value was expected, but missing, and did not count expected absent values as 'missing'. We also excluded the value, 'not done,' from the estimate of the proportion of missing/unknown values for 15 RVCT variables associated with test results.

We computed unbiased estimates for each validation measure and the proportion of missing/unknown values for each variable associated with the statewide cohort, based on the original stratified random sampling design (R survey sampling module version 2.8) [12,13]. For validation measures of non-date variables and missing/unknown values from the RVCT and the medical record, we also computed approximate 95% confidence intervals for proportions, by transforming to a log-odds scale and using the delta method [14]. For dates, we computed the coefficient of variation (C.V.). Analyses were performed using the SAS statistical software package (Release 9.1, SAS Institute Inc., Cary, North Carolina) and R [15].

## Results and discussion

Most reported data submitted to the California TB Registry on the RVCT were highly concordant with data abstracted from the public health medical record. Survey-weighted mean validation measures for 44 categorical variables examined are presented in Table 2. Henceforth, all results are presented as survey-weighted mean estimates for the statewide cohort from which the sample was drawn. Across all categorical variables, concordance ranged from 46.8–100.0%; sensitivity from 26.4–100.0%; and PVP from 1.1–100.0%. Concordance ranged from 84.4–99.0% for demographic data, 91.8–99.1% for social/behavioral risk factor data, 46.8–100.0% for diagnostic and clinical data, and 48.6–100.0% for treatment and case management data. At least 90% of the reported values for 35 (79.5%) categorical variables were identical with values in the medical record. Reported data for the remaining nine (20.5%) categorical variables had lower concordance with the medical record: status of abnormal chest x-ray (46.8%); directly observed therapy (48.6%); smear result for tissue or body fluid other than sputum (49.2%); type(s) of tissue or body fluid for smears and cultures other than sputum (76.4% and 73.9% respectively); provider type (73.4%); occupation (84.4%); sputum culture conversion (85.4%); and sputum smear result (89.6%).

Results for the validity of dates are shown in Table 3. The weighted mean absolute difference in dates in a month-day-year format ranged from 2.6–110.9 days. For dates in a month-year format, the weighted mean absolute difference ranged from 0.7–11.5 months.

The proportion of missing/unknown data for categorical variables and dates for both the RVCT and the medical record is presented in Table 4. Across all variables, the proportion of missing/unknown data on the RVCT ranged from 0.0% to 68.9%, and, for the medical record, the proportion of missing/unknown data ranged from 0.0% to 78.9%. Certain RVCT variables associated with clinical tests have a value of 'not done.' Since the field study did not distinguish between 'not done' and 'not charted' for these variables and recorded both as 'not charted,' a higher proportion of missing/unknown values was noted in the medical record for variables such as results of microscopic smear and culture of other tissue and body fluids.

This is the first study in the U.S. to assess data validity and completeness for the majority of the variables on the RVCT. As the largest single reporter of U.S. TB surveillance data, reporting over 4,000 cases during the 12-month study period, our findings may have direct implications for data quality of the U.S. TB surveillance system. These findings may also be relevant to TB control programs in other U.S. reporting areas or regions of the world. Areas with established TB surveillance systems may benefit from specific findings and evaluation methodology presented [16], while regions with nascent surveillance systems may apply general findings toward the successful development and implementation of surveillance systems.

Our study found generally excellent data validity and completeness for the RVCT variables assessed. From the vantage point of disease control, the most important variables, including initial drug regimen, initial drug susceptibility test results, and patient outcome, were found to be valid. These findings indicate a robust surveillance system that completely and accurately captures essential information to support TB control activities. The nine RVCT variables noted for which data was less concordant with the medical record included demographic, clinical, and case management variables. Data quality for some of these variables may impair program planning and evaluation efforts, especially in the areas of treatment adherence and treatment response, policy development, research, program advocacy, and the allocation of resources for TB control efforts. Variables with potentially the greatest impact on these activities are discussed below in the order of importance.

Ensuring patient adherence to treatment is a core function of TB control programs. Non-adherent patients may be involved in on-going disease transmission and are at increased risk of treatment failure and developing drug-resistant disease [5,17-19]. Directly observed therapy administration (DOT), in which health department personnel watch the patient swallow each dose of medication, is recommended by WHO and CDC to ensure adherence through the required six to nine months of TB treatment (and longer for treatment of multi-drug resistant disease)[1,2,4,20]. Since completion of TB therapy is based on receipt of a prescribed number of doses of a therapeutic regimen, DOT also ensures that patients receive the correct number of doses to complete therapy and achieve cure.

To support the core function of DOT, TB control programs must have valid data with which to evaluate the effectiveness of their DOT activities. RVCT instructions define 'DOT only' as 100% of doses by DOT; 'self-administered therapy only (SAT)' as 0 doses by DOT; and 'both DOT and SAT' as ≥1 DOT dose and ≥1 SAT dose. However, local health departments generally do not implement these strict definitions, which would require them to report therapy given predominantly by DOT or predominantly by SAT as 'both DOT and SAT,' thereby significantly limiting the usefulness of this value. The approach to reporting this variable varies widely by jurisdiction and within jurisdictions (JES personal experience). Concordance between data on the RVCT and the medical record for 'DOT only' was 48.6%. However, the PVP for reported 'DOT only' was 1.1%, indicating that DOT, as defined in the reporting instructions, is rarely provided when it is reported. The PVP for reported 'SAT only,' by comparison, was 88.3%, indicating that 'SAT only' is employed when reported for the vast majority of cases. Thus 'SAT only' may be a more valid indicator of therapy administration. For this reason, many local TB control programs and the state health department in California use 'inappropriate SAT' to evaluate their DOT programs.

The CDC workgroup proposed substantial revisions for this variable, increasing the current three categories to five. Under these proposed revisions, 'total DOT' is defined as 90–100% of doses by DOT, 'predominantly DOT' as 80–89% DOT, and 'predominantly SAT' is defined as 1–49% doses by DOT. The five proposed values correspond to the impact that varying proportions of treatment given by DOT are likely to have on adherence and much better conform to the realities of therapy administration in local health departments. Under this proposed revision, the reported PVP for therapy provided at least predominantly by DOT would improve from 1.1% to 49.9% (not shown). The proposed revised definition of therapy administration is likely to result in substantially more valid and meaningful data for this variable. However, even with these revisions, the challenges local programs face in accurately counting doses received by DOT over a patient's entire treatment may continue to result in inaccurate reporting of the proportion of DOT doses after revised definitions are implemented.

Although CDC recommends universal DOT for TB therapy [4], many California TB control programs do not have sufficient resources to provide universal DOT. In this circumstance, it is recommended that programs prioritize patients for DOT by factors associated with an increased risk of treatment non-adherence and/or the consequences of non-adherence [20]. Drug and alcohol abuse and homelessness figure largely in these factors. Although concordance between data on the RVCT and in the medical record for drug and alcohol abuse ranged from 91.8–98.1%, sensitivity for reported data ranged from 63.5–70.1%. The latter finding indicates that the medical record documented the presence of one or more of these factors approximately one-third more often than was reported on the RVCT.

This review highlighted another issue related to the reporting of these social/behavioral risk factor variables. A relatively high proportion (ranging from 22.1–63.8% for these four variables) of medical records contained no information regarding alcohol use, injection and non-injection drug use, and homelessness (not shown). For medical records that lacked information on these variables, a large proportion of RVCTs did not report 'unknown.' The vast majority of these RVCTs reported that these four factors were explicitly absent (ranging from 76.9–94.7%), rather than present (ranging from 1.3–3.4%) (not shown). Local TB control staff may have procured information regarding these factors outside the medical record and used a paper RVCT only to document this information prior to reporting it. In this circumstance, since data from paper RVCTs in the medical record were not abstracted by the study team, more complete information for these variables may be contained on the RVCT than the abstracted medical record. However, it is unlikely that such a high proportion of these variables, in comparison to other variables, were differentially reported only on a paper RVCT, without other documentation in the medical record. It is also possible that the absence of documentation regarding these factors in the medical record was taken as evidence of their absence by local reporting staff and others. During data abstraction, approximately one-sixth of the medical records that had missing information for drug and alcohol abuse and homelessness were documented as 'no,' rather than 'not charted.' Thus, an even greater proportion of medical records did not contain information on these important factors associated with treatment non-adherence.

Accurate and complete elicitation and reporting of substance abuse and homelessness should be a high priority to ensure that patients with these factors are identified and prioritized for DOT and other adherence-promoting strategies. These core risk factors should be part of standard intake forms for suspected TB patients. In addition to DOT, homeless patients and those with substance abuse often require more complex case management [5,17]. For this reason, CDC's revised funding formula, implemented in January 2005 (K. Castro, Director, CDC Division of Tuberculosis Elimination, letter to colleagues; October 15, 2004), increased funding for cases with these and other characteristics. Programs receive less federal funding than warranted if information about homelessness and substance abuse within the year prior to diagnosis is not accurately elicited from patients and reported.

Another factor that may be associated with non-adherence to treatment is a prior episode of TB disease, which also increases the risk of drug-resistant TB disease [5,17-19]. Thus, it is a high priority to identify patients with previous TB to ensure that they receive DOT and an appropriate initial drug regimen. Information about a prior episode of TB is also important to distinguish between primary and acquired drug-resistant TB, which should lead to very different TB control strategies. Although 94.6% of RVCT data for previous TB was concordant with the medical record, sensitivity for reported previous TB was 57.6%, indicating that previous TB was documented in the medical record over 40% more often than it was reported on the RVCT. Furthermore, it is of note that almost one-fifth of medical records lacked any information regarding previous TB. However, unlike other categorical variables on the RVCT, the paper RVCT restricted values for previous TB to 'yes' and 'no,' while the electronic form permitted the entry of 'unknown.' If local health departments use the paper RVCT or similar forms, based on the RVCT, to capture information from the medical record for computer data entry, none of the cases with missing information for previous TB in the medical record could be correctly reported as 'unknown.' In the reporting of previous TB that was not documented in the medical record, an estimated 2.2% (11) of the statewide cohort was reported with previous TB versus 97.8% (509) reported without previous TB (not shown). This may result in an underestimate of cases with previous TB. From the standpoint of data quality, the electronic information system should capture the same values as the paper form for each RVCT variable, and variables should be structured to permit reporting of 'unknown' values.

The time to documented sputum culture conversion (conversion of an initially positive *M. tuberculosis *sputum culture to a consistently negative result) is an important indicator of a patient's response to therapy. As such, timely sputum culture conversion is an important evaluation measure for TB control programs. If delayed sputum culture conversion (conversion >60 days) is noted, the following interventions are recommended: assessment of factors potentially associated with suboptimal response to treatment, extended duration of TB therapy, and initiation of DOT (if not already provided) [20]. Reported data for documentation of sputum culture conversion were 85.4% concordant with data in the medical record, and the PVP for a reported positive was 87.8%, indicating that greater than 10% of cases reported with sputum culture conversion lacked documentation in the medical record. For cases with documented sputum culture conversion, the absolute mean difference between the RVCT and medical record in the date for the first consistently negative sputum culture was 25.0 days. The magnitude of this difference is almost half of the timeframe (60 days) recommended to trigger interventions for delayed sputum culture conversion noted above. Complete and accurate data validity for these variables is essential for TB control programs to evaluate their performance in achieving timely sputum culture conversion. This performance measure highlights medical oversight, case management, and laboratory functions. Inadequate data impairs the ability of programs to identify and address problems in these important areas, to prevent on-going transmission from patients who remain sputum culture positive, and to ensure cure of TB patients.

RVCT data for cases with an initial abnormal chest x-ray were only 46.8% concordant with the medical record in assessing chest x-ray status (stable, improving, or worsening) of a follow-up chest film. The poor concordance has multiple possible explanations: unclear RVCT instructions regarding the timeframe from the initial to the follow-up chest x-ray; follow-up chest x-ray results received after the RVCT was submitted; multiple chest x-ray results with potentially differing values; and inadequate correspondence between RVCT values and terms used in chest x-ray reports. The variable, 'chest x-ray status,' is used primarily to execute an algorithm to verify that patients without positive *M. tuberculosis *cultures have TB disease [21]; since only culture positive cases were included in the study sample, validity of this variable is not relevant for cases in the study population. But poor concordance between RVCT data and the medical record suggests that using a reported change in chest x-ray status as a criterion to verify culture-negative TB cases may be problematic. A high proportion of data for this variable was also missing/unknown from both the medical record and the RVCT (45.3% and 68.9%, respectively). The CDC workgroup proposed to eliminate this variable entirely from the RVCT; poor data validity and a high proportion of missing data in our study support this proposal.

As noted, data for the smear result of tissue or body fluid other than sputum were 49.2% concordant. On both the RVCT and the medical record, at least one tissue or body fluid type was associated with each positive smear result, with 76.4% concordance on tissue/body fluid types(s). Among discordant results for positive smear, 54.4% were associated with a specific tissue/body fluid type that was documented in the medical record, but not reported on the RVCT (not shown). Conversely, the RVCT reported 45.6% of positive smear results associated with a specific tissue or fluid that was not documented in the medical record (not shown). Local TB control staff may have received telephone reports of these results and documented them only on a paper RVCT in the medical record before reporting it. In this circumstance, since the study team did not abstract data from paper RVCTs in the medical record, more complete information on these variables for some cases may be contained on the RVCT than the abstracted medical record. Results that were documented in the medical record, but were not reported may be explained by the retrospective nature of the field study which benefited from results and documentation that may not have been available at the time the RVCT was submitted. Data on microscopic smear results for tissue or body fluid other than sputum are used for TB case confirmation among a very small proportion (approximately 0.5%) of TB cases who lack sputum and/or other cultures to confirm TB [21]. Since all cases in the sample were *M. tuberculosis *culture positive, discordance of data between the RVCT and the medical record for these variables has little consequence for the study population. Among patients whose TB case confirmation relies on microscopic smear results, the absence of these data could delay case confirmation and reporting. The proposed RVCT revision provides much more detailed and specific instructions for reporting and updating information for these variables that will likely improve data quality.

Both local and state TB control programs often stratify program performance measures by provider type to better understand the role of public versus private provider in the measurement of interest and to inform interventions. Our study found that RVCT data for provider type was 73.4% concordant with the medical record. These findings are likely the result of instructions and categories that do not accurately reflect the range and complexity of provider types involved in the direct care of patients with an illness lasting 6–18 months. Proposed revisions of the CDC workgroup would expand categories for provider type, clarify definitions and instructions, and allow reporting of as many provider types that apply.

RVCT data for case occupation in the year prior to TB diagnosis were 84.4% concordant with data in the medical record. The sensitivity for migratory agricultural worker was 51.8%, indicating that nearly twice as many of these workers were documented in the medical record but not reported on the RVCT. The sensitivity for 'other employment' (employment other than migratory agricultural worker, health care worker, and correctional employee) was 76.4%. The vast majority of patients who were identified with other employment in the medical record were reported as 'unemployed' on the RVCT (not shown). The CDC workgroup proposed to expand and redefine occupational categories and clarify instructions for reporting case occupation.

Turning from the categorical variables to the nine date variables we compared, we found that for dates in month/day/year format, the weighted mean absolute difference in days between the medical record and the report was generally within 25 days, except for birth date which had a difference of 48 days. However, any difference may be problematic when a variable, such as birth date, is used to match cases in surveillance datasets. For foreign-born cases, the weighted mean difference between the date of entry to the U.S. (in month/year format) on the RVCT and in the medical record was 11.2 months. Such discordance may be related to recall bias of the TB patient and may be difficult to rectify. Alternatively, it may be related to unclear reporting instructions principally affecting patients with repeated entries to the U.S. or with different countries of birth from their countries of origin.

With notable exceptions, across all variables, the proportion of missing data on the RVCT was lowest for demographic variables, drug susceptibility test results, and the initial treatment regimen. Across all variables, the mean proportion of missing/unknown data was greater in the medical record (9.8%) versus the RVCT (2.9%), including data noted above for drug and alcohol use, homelessness, and previous TB. However, it should be noted that 15 RVCT variables pertaining to clinical tests have a 'not done' value, which was abstracted as 'not charted' in the field study. Missing data potentially compromise the quality of surveillance data and the surveillance system, especially if data are not missing completely at random. In the latter circumstance, biases may be introduced that impair our understanding of the true epidemiology of TB and compromise activities that rely on accurate data.

The study findings provide strong support of the CDC workgroup's proposed revisions to the 1993 RVCT, including restructuring certain variables (e.g., previous TB, DOT, type of health care provider); enhanced reporting instructions for other variables (e.g., proposed instructions specify submission of updated information as it becomes available for drug and alcohol abuse, and smear and culture results of tissue and other body fluids); clarification of instructions for some date fields (e.g., date of entry to the U.S. for foreign-born cases); and deletion of one variable (e.g., chest x-ray status).

Implementation of the proposed RVCT is likely to make a substantial contribution to improving the validity and value of TB surveillance data. However, additional interventions are needed to ensure valid TB surveillance data nationally. As more and more health departments implement comprehensive electronic patient management systems, it is important for the accuracy of the RVCT generated by these systems to be fully evaluated. These systems should be designed to ensure that definitions and values for RVCT variables are consistent nationally and reinforced by the functionality of the system so that the information needed to support and sustain our TB control activities is accurately captured.

Although CDC has provided instructions for completing the RVCT, detailed documentation regarding electronic reporting, and extensive electronic data validation, it has not provided guidance on effective data QC practices at the local level. An informal survey of many California health departments revealed that nearly half did not have processes in place to check the completed RVCT against the medical record, and 80% did not check for data entry errors [22]. Since it is likely that gaps in QC practices exist broadly across the nation and there are numerous generally applicable QC practices for an established TB surveillance system, it is appropriate for the CDC to address these deficiencies and implement enhanced QC practices for the surveillance system. In order to ensure data quality in surveillance systems, development of best practices for recordkeeping and documentation in the public health medical record is also needed. As public heath programs and bioterrorism preparedness entities move toward electronic surveillance and patient management systems, the importance of electronic data quality in effective patient management cannot be overemphasized.

To improve and ensure RVCT data quality as CDC implements the revised RVCT, targeted interventions at the national, state, and local levels are also needed. Potential interventions include the following:

▪ National level

     ▪ Field test proposed RVCT revisions and reporting instructions prior to their implementation

     ▪ Develop recommendations and tools for QC practices at the local level that facilitate implementation of effective practices

     ▪ Develop best practices and tools for recordkeeping and documentation to support accurate and complete capture of data for RVCT variables

     ▪ Develop and maintain a TB surveillance users group to provide on-going training and capacity-building of TB registry staff nationally

▪ State level

     ▪ Provide regular training to local TB control staff who complete and enter the RVCT to ensure that staff understand definitions and instructions and appreciate the importance of data quality

     ▪ Modernize and centralize user-assistance resources (i.e., Help functions) either directly within new information systems or via the World Wide Web to ensure access and uniform distribution of revisions

     ▪ Provide technical assistance to support implementation of QC practices by local staff

▪ Local level

     ▪ Expand use of data QC procedures

     ▪ Implement effective recordkeeping and documentation in the public health medical record to support complete and accurate capture of data for RVCT variables

     ▪ Routinely update case reports as more current case information becomes available

     ▪ Ensure that replacement or upgraded information systems can capture TB surveillance data according to national requirements

There are several limitations to this study. First, the study population is drawn from cases counted during 6/1/96-5/31/97. However, significant statewide interventions to improve data quality have not been undertaken since 1997, nor have the noted structural problems with RVCT variables or unclear reporting instructions been resolved. For these reasons, it is likely that findings of the study substantially reflect data quality at the present time. Second, the findings may not be representative of RVCT data validity in other states or in other health jurisdictions in California. Third, the field study was not primarily designed to validate the RVCT; some definitions in the field study do not exactly match RVCT definitions (e.g., history of homelessness), and a small number of RVCT variables were not abstracted from the medical record so that they could not be validated. Fourth, the medical record may be an imperfect "gold standard", and there may be situations in which more accurate and complete information is reported than is contained in the medical record. Fifth, abstractors coded approximately one-sixth of medical records that lacked documentation for homelessness and substance abuse as explicitly lacking these factors. This may have resulted in an underestimate of the relatively high proportion of RVCTs that recorded the absence of these factors, with no documentation in the medical record. Sixth, the estimates for sputum smear and sputum culture were less robust than for other variables because of the way that data was abstracted in the field study. In assessing the validity of these two variables, we limited the comparison to patients with pulmonary TB only without non-sputum specimen results.

In closing the discussion on limitations, several interpretive comments regarding the validation measures are in order. In general, concordance is most appropriate in assessing the level of agreement between two equivalent data sources, rather than a primary and secondary data source. Moreover, because concordance does not account for agreement that occurs by chance alone, our estimates of concordance should be considered 'high' estimates for measures of data validity. Similarly, estimates for sensitivity of the reported positive value should also be seen as 'high' estimates, since we restricted our calculations to 'yes' and 'no' values on the RVCT and medical record. In fact, if the medical record contained documentation of a positive value that was reported on the RVCT as 'unknown' or 'not done,' our estimates would have excluded these discordant values, thereby resulting in a higher estimate of sensitivity.

## Conclusion

This evaluation provides unique information on the validity of TB surveillance data in a large national public health disease surveillance system. Since California has contributed over 20% of U.S. TB cases annually over the last decade, these findings may have implications for the validity of TB surveillance data nationally. Collection of valid TB surveillance data is critical to the public's health. Invalid data may have unintended consequences, including impaired understanding of the true epidemiology of TB; compromised program planning, evaluation, and research; inaccurate targeting of interventions; inadequate TB prevention and control policies; and inappropriate allocation of resources.

Our study found that data validity and completeness for most reported variables were excellent, indicating a robust surveillance system. However, lower data validity was noted for nine categorical (20.5%) variables, with the most important impacts on evaluation of DOT, a core component in TB control programs to ensure treatment adherence and cure. In addition, it is likely that some of the factors (e.g., homelessness, substance abuse, and previous TB) on which TB control programs prioritize patients for DOT may not be captured accurately and completely by the RVCT, further compromising DOT activities and our evaluation of them.

The study findings provide compelling evidence in support of the CDC workgroup's proposed revisions of the TB case report. The proposed enhanced instructions and definitions, revisions to variable structures, and the addition of new variables are likely to contribute substantially both to improved data quality and more useful data. Since the RVCT remains under revision and will undergo stakeholder review before it is finalized, additional revisions are anticipated. Regardless, greater attention to QC and recordkeeping at the national, state, and local levels will help to ensure that implementation of the revised RVCT achieves maximum impact.

## Competing interests

The author(s) declare that they have no competing interests.

## Authors' contributions

JES conceived the study, participated in study design and data analysis, and drafted the manuscript.

ESL conducted original field study, including data collection and data management, participated in study design and drafting of manuscript.

TCP participated in data analysis and review of manuscript drafts.

JMF participated in study design and review of manuscript drafts.

JLW coordinated the California TB Registry, participated in study design and review of manuscript drafts.

All authors read and approved the final manuscript.

## Pre-publication history

The pre-publication history for this paper can be accessed here:



## Figures and Tables

**Table 1:** Summary of variables and validation measures estimated in the medical record and case report

**Variable Type** **(n, %)**		Variable Number*	**Medical Record Values**	**Report of Verified Case of Tuberculosis (RVCT) Values**	**Number of Values and Validation Measures Estimated**
	n = 34 (77%)	1, 3, 4, 7, 8, 9, 10, 11, 12, 13, 15, 16, 17, 19, 21, 22, 24, 25, 26, 27, 28, 29, 30, 31, 32, 33, 34, 35, 39, 40, 41, 42, 43, 44	'Yes' 'No' 'Not Charted'	'Yes' 'No' 'Not Done' (n = 15) 'Unknown'	2 × 2 tables: Concordance Sensitivity Positive Predictive Value
	
Categorical n = 44		2, 6, 14, 23, 36, 37, 38	Various values	Various values	n × n tables (values = 3–6): Concordance
	n = 10 (23%)				2 × 2 tables: collapsed n × n tables to n (2 × 2) tables^†^
					Sensitivity Positive Predictive Value
		
		5, 18, 20			n × n tables (values=96–268): Concordance

Dates n = 9	n = 7 (78%)	Month/Day/Year 46, 48, 49, 50, 51, 52, 53	MM/DD/YYYY	MM/DD/YYYY	Absolute mean difference (days)
	
	n = 2 (22%)	Month/Year 45, 47	MM/DD/YYYY	MM/YYYY	Absolute mean difference (months)

* See Table 2, first column, for variable number (*Note*: these numbers do not correspond to the variable numbers on the RVCT form).

† For each of the n values, we constructed a 2 × 2 table from (1) the number of individuals for which the medical record and RVCT both exhibited that value, (2) the number for which only the medical record exhibited that value, (3) the number for which only the RVCT exhibited that value, and (4) the number for which neither exhibited that value. Specifically, for each *k *(*k *= 1, ..., *n*), we constructed a collapsed 2 × 2 table of values

xij(k)
 MathType@MTEF@5@5@+=feaafiart1ev1aaatCvAUfKttLearuWrP9MDH5MBPbIqV92AaeXatLxBI9gBaebbnrfifHhDYfgasaacH8akY=wiFfYdH8Gipec8Eeeu0xXdbba9frFj0=OqFfea0dXdd9vqai=hGuQ8kuc9pgc9s8qqaq=dirpe0xb9q8qiLsFr0=vr0=vr0dc8meaabaqaciaacaGaaeqabaqabeGadaaakeaacqWG4baEdaqhaaWcbaGaemyAaKMaemOAaOgabaGaeiikaGIaem4AaSMaeiykaKcaaaaa@341B@ (*i *= 1, 2; *j *= 1, 2) according to  for each *k *= 1, ..., n. with *a*_*lm *_being the values in the original n × n table (*l *= 1, ..., *n*, *m *= 1, ...*n*). (Here, *δ*_*ab *_is the Kronecker delta, i.e., *δ*_*ab *_= 1 if a = b and 0 otherwise.)

**Table 2:** Weighted mean validation measures for categorical variables in Report of Verified Case of Tuberculosis (RVCT)

#*	**RVCT Variable**	**Concordance (95% CI**^†^**) %**		**Sensitivity of Reported Value (95% CI) %**		**Predictive Value of Reported Positive Value (95% CI) %**	
	**DEMOGRAPHIC**						
1	Sex	98.1	(96.7, 99.0)	99.7	(98.0, 99.9)	97.5	(95.3, 98.6)
2	Race	97.7	(96.3, 98.6)	ND^‡^	ND	ND	ND
	White	ND	ND	99.2	(97.5, 99.7)	97.0	(94.4, 98.4)
	Black	ND	ND	95.3	(87.1, 98.4)	97.9	(88.8, 99.7)
	Asian-Pacific Islander	ND	ND	97.5	(94.9, 98.8)	98.4	(96.3, 99.3)
	American Indian/Alaska Native	ND	ND	50.0	(12.8, 87.2)	100.0	-^§^
3	Ethnicity	98.5	(97.2, 99.1)	97.6	(94.7, 98.9)	98.1	(95.5, 99.2)
	Country of origin						
4	U.S.-born	98.9	(97.7, 99.4)	99.1	(95.1, 99.9)	96.6	(92.8, 98.4)
5	Other country of origin	96.0	(93.6, 97.5)	ND	ND	ND	ND
6	Occupation	84.4	(80.7, 87.5)	ND	ND	ND	ND
	Health care worker	ND	ND	78.2	(46.4, 93.7)	70.5	(41.3, 89.0)
	Migratory agricultural worker	ND	ND	51.8	(25.1, 77.5)	81.1	(38.3, 96.7)
	Other occupation	ND	ND	76.4	(69.8, 81.9)	89.0	(83.3, 92.9)
	Not employed within past 24 months	ND	ND	93.5	(89.5, 96.0)	82.2	(76.9, 86.5)
7	Resident of long-term care facility at diagnosis	98.3	(97.1, 99.0)	67.8	(47.1, 83.3)	76.8	(57.8, 88.9)
8	Resident of correctional facility at diagnosis	99.0	(97.7, 99.6)	69.7	(45.7, 86.3)	100.0	-
	**SOCIAL AND BEHAVIORAL RISK FACTORS**						
9	Homeless within past 1–2 years^||^	99.1	(96.9, 99.7)	97.3	(92.6, 99.0)	95.1	(75.9, 99.2)
10	Injecting drug user within past year	98.1	(96.5, 99.0)	63.5	(41.5, 81.0)	86.5	(59.8, 96.5)
11	Non-injecting drug user within past year	96.6	(94.6, 97.9)	70.1	(55.2, 81.7)	90.9	(74.6, 97.1)
12	Excess alcohol use within past year	91.8	(88.8, 94.0)	69.9	(61.0, 77.6)	96.8	(89.3, 99.1)
	**DIAGNOSTIC AND CLINICAL**						
13	Previous TB	94.6	(92.3, 96.2)	57.6	(45.0, 69.3)	93.5	(81.9, 97.9)
14	Site of disease	92.5	(90.4, 94.2)	ND	ND	ND	ND
	Pulmonary only	ND	ND	96.7	(94.6, 97.9)	96.2	(94.3, 97.4)
	Extra-pulmonary only	ND	ND	98.6	(96.8, 99.4)	81.6	(74.4, 87.0)
	Both pulmonary and extra-pulmonary	ND	ND	51.1	(39.7, 62.4)	86.9	(73.9, 94.0)
15	Sputum smear	89.6	(86.0, 92.3)	90.5	(85.8, 93.7)	92.2	(87.9, 95.1)
16	Sputum culture	96.8	(94.2, 98.2)	100.0	-	96.8	(94.2, 98.2)
17	Smear of tissue/body fluids (non-sputum)	49.2	(38.4, 60.0)	31.2	(21.0, 43.6)	98.7	(98.1, 99.2)
18	Tissue/body fluid type (non-sputum)	76.4	(59.3, 87.8)	ND	ND	ND	ND
19	Culture of tissue/body fluid (non-sputum)	98.2	(94.6, 99.4)	100.0	-	98.2	(94.6, 99.4)
20	Tissue/body fluid type (non-sputum)	73.9	(66.0, 80.6)	ND	ND	ND	ND
	Chest x-ray						
21	Abnormal/normal	96.0	(94.0, 97.3)	99.1	(97.6, 99.7)	96.8	(94.8, 98.0)
22	If abnormal: cavitary/non-cavitary**	90.9	(88.0, 93.2)	75.1	(66.3, 82.3)	86.2	(78.0, 91.7)
23	If abnormal: stable/worsening/improving	46.8	(37.0, 56.9)	ND	ND	ND	ND
	Stable	ND	ND	49.9	(36.0, 63.7)	53.7	(39.0, 67.9)
	Worsening	ND	ND	52.0	(38.0, 65.7)	51.7	(37.8, 65.4)
	Improving	ND	ND	26.9	(8.7, 58.6)	19.8	(6.5, 46.8)
24	Initial drug susceptibility testing done	100.0	-	100.0	-	100.0	-
	Results of initial drug susceptibility testing^††^						
25	Isoniazid	99.2	(98.1, 99.7)	92.5	(82.6, 97.0)	100.0	-
26	Rifampin	100.0	-	100.0	-	100.0	-
27	Pyrazinamide	92.1	(89.3, 94.2)	88.1	(68.8, 96.1)	100.0	-
28	Ethambutol	100.0	-	100.0	-	100.0	-
29	Streptomycin	98.3	(96.8, 99.1)	93.2	(78.8, 98.0)	92.0	(78.7, 97.3)
	**TREATMENT AND CASE MANAGEMENT**						
	Initial treatment regimen						
30	Isoniazid	98.2	(96.8, 99.0)	99.6	(98.8, 99.9)	98.6	(97.2, 99.3)
31	Rifampin	98.7	(97.5, 99.4)	99.9	(99.7, 100.0)	98.8	(97.6, 99.4)
32	Pyrazinamide	96.7	(95.1, 97.8)	98.1	(96.7, 98.9)	98.4	(97.1, 99.1)
33	Ethambutol	95.9	(94.0, 97.2)	98.8	(97.4, 99.4)	96.5	(94.6, 97.8)
34	Streptomycin	98.7	(97.7, 99.3)	40.3	(16.3, 70.1)	38.1	(14.1, 69.9)
35	Sputum culture conversion documented	85.4	(81.1, 88.8)	93.3	(89.5, 95.7)	87.8	(83.0, 91.3)
36	Reason therapy stopped^‡‡^	97.9	(96.4, 98.8)	ND	ND	ND	ND
	Completion of therapy	ND	ND	97.8	(96.0, 98.8)	100.0	-
	Lost/uncooperative or refused therapy	ND	ND	98.4	(98.4, 98.4)	62.9	(46.1, 77.1)
	Died	ND	ND	99.2	(96.6, 99.8)	93.3	(84.2, 97.4)
37	Provider type	73.4	(69.8, 76.7)	ND	ND	ND	ND
	Health Department only (HD)	ND	ND	74.7	(69.7, 79.1)	90.3	(86.7, 93.0)
	Private/Other only	ND	ND	79.6	(74.6, 83.9)	80.0	(74.2, 84.8)
	Both HD and Private/Other	ND	ND	38.6	(26.4, 52.5)	18.1	(12.0, 26.4)
38	Therapy administration	48.6	(44.4, 52.8)	ND	ND	ND	ND
	Directly Observed Therapy (DOT) only	ND	ND	29.7	(10.5, 60.4)	1.1	(0.4, 2.9)
	Self-administered Therapy (SAT) only	ND	ND	72.0	(66.7, 76.7)	88.3	(83.1, 92.0)
	Both DOT and SAT	ND	ND	26.4	(21.3, 32.2)	82.7	(73.1, 89.4)
39	Final drug susceptibility testing done ^‡‡^	100.0	-	100.0	-	100.0	-
	Results of final drug susceptibility testing^††§§^						
40	Isoniazid	100.0	-	100.0	-	100.0	-
41	Rifampin	98.4	(97.3, 99.0)	92.6	(79.7, 97.5)	100.0	-
42	Pyrazinamide	90.5	(59.5, 98.4)	-	-	-	-
43	Ethambutol	90.3	(59.0, 98.4)	-	-	-	-
44	Streptomycin	100.0	-	100.0	-	100.0	-

*Note*: All results are presented as survey-weighted mean estimates for the statewide cohort from which the sample was drawn.

* Variable number (see Table 1)

† Confidence interval

‡ Not determined, by design

§ No value computed (e.g., division by 0)

|| RVCT defines homeless within 1 year of diagnosis; medical record review defined homeless within 2 years of diagnosis

** RVCT values: cavitary, non-cavitary consistent with TB, non-cavitary not consistent with TB; medical record review values: cavitary, abnormal/non-cavitary

†† As a result of a very small number of second-line drug susceptibility test results, these results are not presented.

‡‡ Excludes 9 cases: 1 case without TB; 5 cases reported as 'moved' on RVCT; 3 cases reported as 'other" on RVCT

§§ Based on small number of cases with final susceptibility testing performed; therefore estimates are subject to considerable instability

**Table 3:** Weighted absolute mean difference between dates on the case report and the medical record

**Dates**	**Weighted Absolute Mean Difference (days)**	**Coefficient of Variation (C.V.) %**
*Month/day/year dates*		
Birth date	48.4	39.9
Treatment start	2.6	17.0
Isolate collection for drug susceptibility testing	13.0	26.1
First positive culture	6.1	31.0
First consistently negative culture	25.0	15.1
Treatment stop	19.2	11.3
Isolate collection for final susceptibility testing	110.9*	44.5

**Dates**	**Weighted Absolute Mean Difference (months)**	**Coefficient of Variation (C.V.) %**

*Month/year dates*		
Case reported to local health department	0.74	10.0
Entry into U.S.	11.5	20.2

*Note*: All results are presented as survey-weighted mean estimates for the statewide cohort from which the sample was drawn.

* Based on small number of cases with final susceptibility testing performed; therefore estimate is subject to considerable instability

**Table 4:** Weighted mean estimates for the proportion of missing/unknown values on case report and medical record

#*	**Report of Verified Case of Tuberculosis (RVCT) Variable**	**RVCT Missing/Unknown % (95% CI)**^†^		**Public Health Medical Record Missing/Unknown % (95% CI)**	
	**DEMOGRAPHIC**				
1	Sex	0.0	-^‡^	0.0	-
2	Race	0.0	-	1.3	(0.7, 2.6)
3	Ethnicity	0.0	-	1.4	(0.7, 2.6)
	Country of origin				
4	U.S.-born	0.6	(0.2, 1.5)	2.7	(1.7, 4.2)
5	Other country of origin	0.0	-	0.1	(0.1, 0.1)
6	Occupation	8.2	(6.3, 10.6)	23.6	(20.4, 27.1)
7	Resident of long-term care facility at diagnosis	0.2	(0.1, 0.4)	7.6	(5.8, 9.9)
8	Resident of correctional facility at diagnosis	0.1	(0.0, 0.3)	6.7	(5.1, 8.9)
	**SOCIAL/BEHAVIORAL RISK FACTORS**				
9	Homeless within past 1–2 years^§^	1.9	(1.1, 3.2)	63.8	(59.9, 67.5)
10	Injecting drug user within past year	8.0	(6.2, 10.2)	22.1	(19.1, 25.4)
11	Non-injecting drug user within past year	7.8	(6.0, 10.0)	23.4	(20.3, 26.8)
12	Excess alcohol use within past year	7.6	(5.9, 9.8)	24.1	(21.0, 27.6)
	**DIAGNOSTIC AND CLINICAL**				
13	Previous TB	0.0	-	19.7	(16.8, 22.9)
14	Site of disease	0.0	-	0.0	-
15	Sputum smear^||^	4.9	(3.4, 7.2)	6.0	(4.2, 8.7)
16	Sputum culture^||^	15.1	(12.1, 18.7)	14.4	(11.5, 17.8)
17	Smear of tissue/body fluids (non-sputum)^||^	0.6	(0.3, 1.5)	78.9	(75.6, 82.0)
18	Tissue/body fluid type (non-sputum)* *	0.0	-	0.0	-
19	Culture of tissue/other body fluid (non-sputum)^||^	0.3	(0.1, 1.1)	63.6	(59.8, 67.2)
20	Tissue/body fluid type (non-sputum) * *	0.0	-	0.0	-
	Chest x-ray results				
21	Abnormal/normal^||^	0.6	(0.3, 1.5)	8.7	(6.8, 11.2)
22	If abnormal: cavitary/non-cavitary**	0.1	(0.0, 0.4)	0.0	-
23	If abnormal: stable/worsening/improving**	68.9	(64.8, 72.7)	45.3	(40.9, 49.7)
24	Initial susceptibility testing done	0.0	-	1.7	(0.9, 3.1)
	Results of initial susceptibility testing, if done^††^				
25	Isoniazid^||^	0.0	-	3.1	(2.0, 4.9)
26	Rifampin^||^	0.7	(0.3, 1.8)	21.2	(18.1, 24.5)
27	Pyrazinamide^||^	0.0	-	3.1	(2.0, 4.9)
28	Ethambutol^||^	0.0	-	4.1	(2.8, 6.0)
29	Streptomycin^||^	0.0	-	2.9	(1.8, 4.6)
	**TREATMENT AND CASE MANAGEMENT**				
	Initial treatment regimen				
30	Isoniazid	0.0	-	0.0	-
31	Rifampin	0.0	-	0.0	-
32	Pyrazinamide	0.1	(0.0, 0.3)	0.0	-
33	Ethambutol	0.0	-	0.0	-
34	Streptomycin	0.1	(0.0, 0.3)	0.0	-
35	Sputum culture conversion, if ever sputum culture positive**	1.6	(0.7, 3.5)	0.0	-
36	Reason therapy stopped	0.7	(0.3, 1.5)	0.0	-
37	Provider type	0.2	(0.0, 1.3)	0.0	-
38	Therapy administration	3.5	(2.4, 5.2)	0.0	-
39	Final susceptibility testing done^‡‡^	0.0	-		
	Results of final susceptibility testing, if done^‡‡^				
40	Isoniazid^||‡‡^	0.0	-	5.7	(1.5, 19.6)
41	Rifampin^||‡‡^	0.0	-	5.7	(1.5, 19.6)
42	Pyrazinamide^||‡‡^	5.1	(0.9, 25.2)	11.9	(4.9, 26.2)
43	Ethambutol^||††^	0.0	-	6.4	(1.9, 19.3)
44	Streptomycin^||‡‡^	0.0	-	9.8	(3.3, 25.4)
	**DATES**				
45	TB case report date	0.0	-	5.7	(4.1, 7.8)
46	Date of birth	0.0	-	0.1	(0.0, 0.3)
47	Date arrived in United States (foreign-born cases)**	9.2	(7.0, 12.1)	15.1	(12.1, 18.6)
48	Treatment start date	0.0	-	0.0	-
49	Date specimen collected for initial drug susceptibility testing**	0.0	-	2.1	(1.2, 3.7)
50	Date specimen collected on initially positive sputum culture**	0.0	-	0.0	-
51	Date specimen collected on first consistently negative sputum culture**	1.4	(0.5, 3.8)	0.2	(0.2, 0.2)
52	Date TB treatment stopped	0.2	(0.2, 0.2)	0.0	-
53	Date specimen collected for final drug susceptibility testing^††‡‡^	5.1	(0.9, 25.2)	9.8	(3.3, 25.4)

*Note*: All results are presented as survey-weighted mean estimates for the statewide cohort from which the sample was drawn.

* Variable number (see Table 1)

† Confidence Interval

‡ With an estimate of 0.0% missing/unknown, a confidence interval was not determined

§ RVCT defines homeless within 1 year of diagnosis; medical record review defined homeless within 2 years of diagnosis

|| 'Not done' on RVCT excluded

** For nine RVCT variables, values are expected depending on the value of another variable (e.g., a value for type of chest x-ray abnormality or status of chest x-ray is expected only if chest x-ray results are abnormal). For these nine variables, we restricted our estimate of missing/unknown values to instances in which a subsequent value was expected, but missing, and did not count expected absent values as 'missing'.

†† As a result of a very small number of second-line drug susceptibility test results, these results are not presented, either for initial or final drug susceptibility testing.

‡‡ Based on small number of cases with final susceptibility testing performed; therefore estimate is subject to considerable instability
